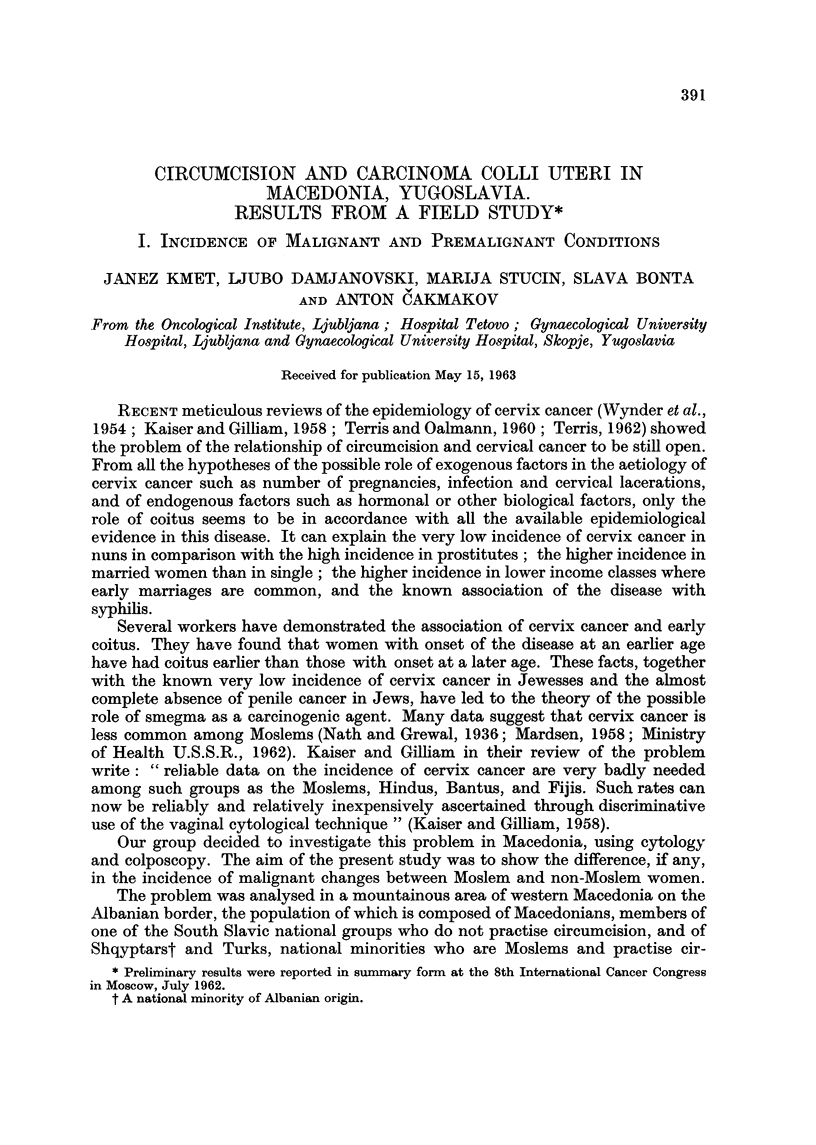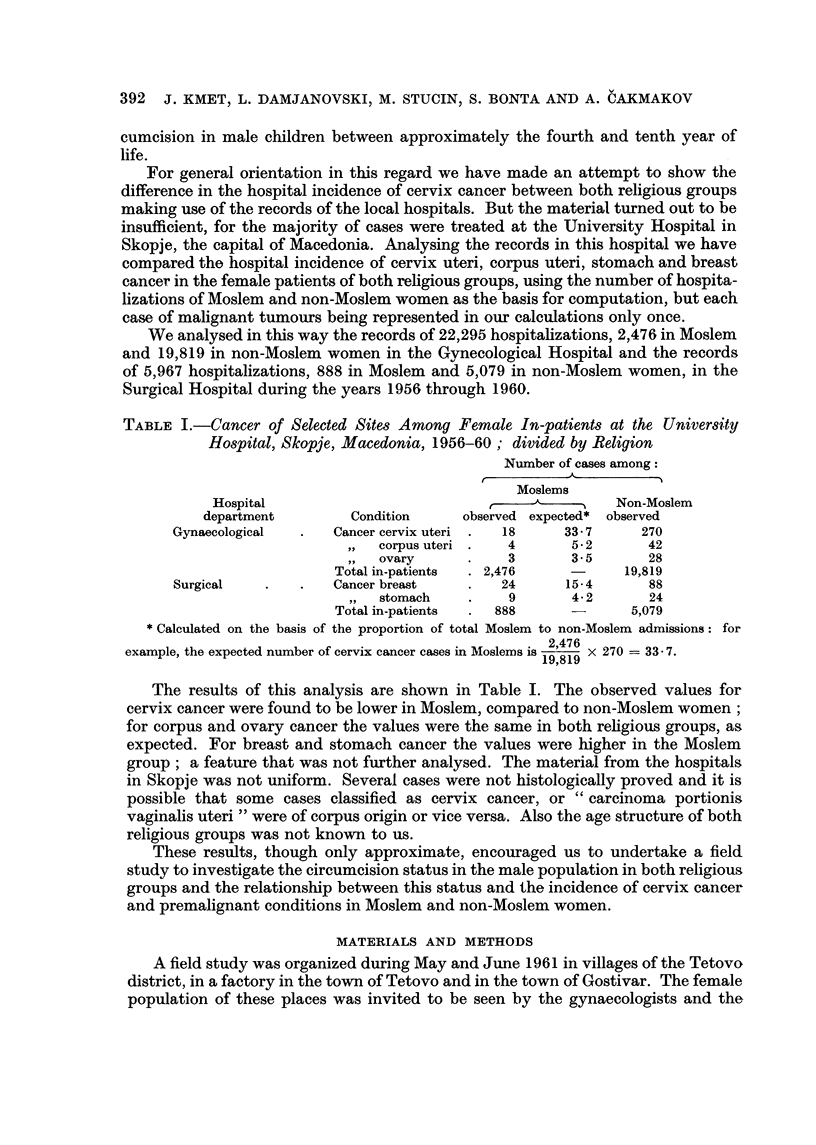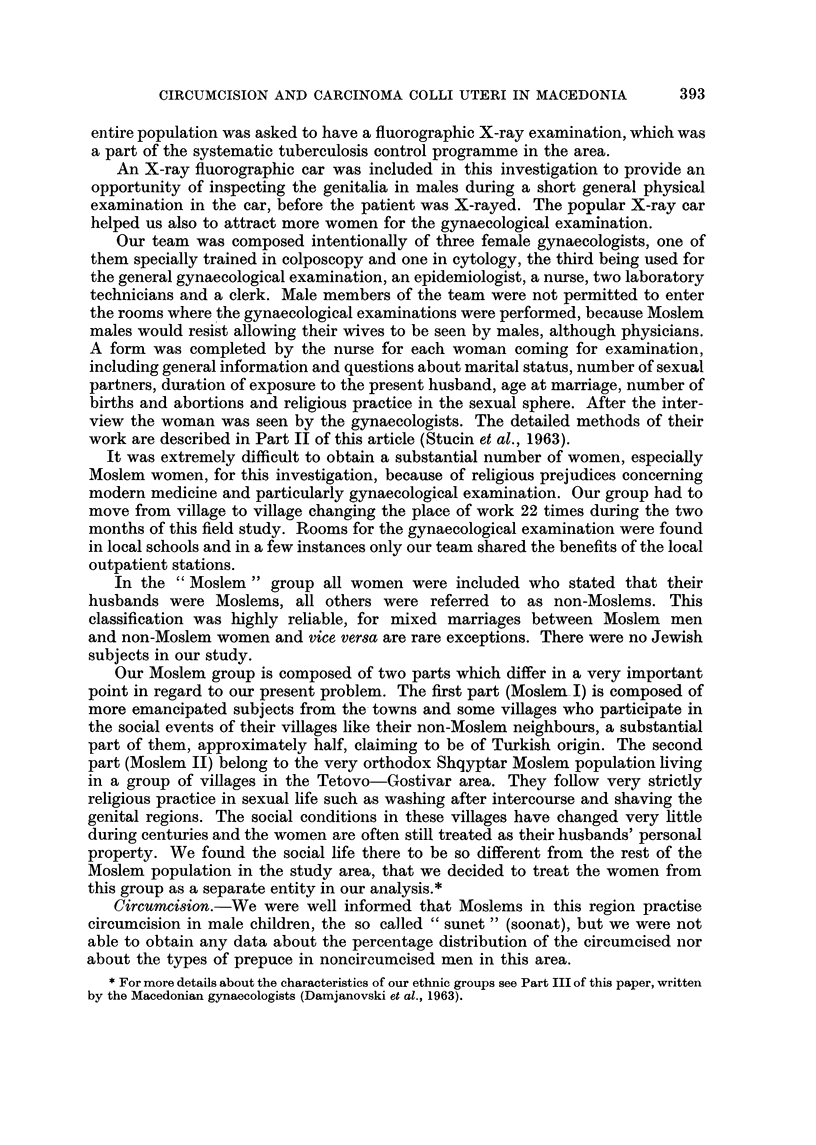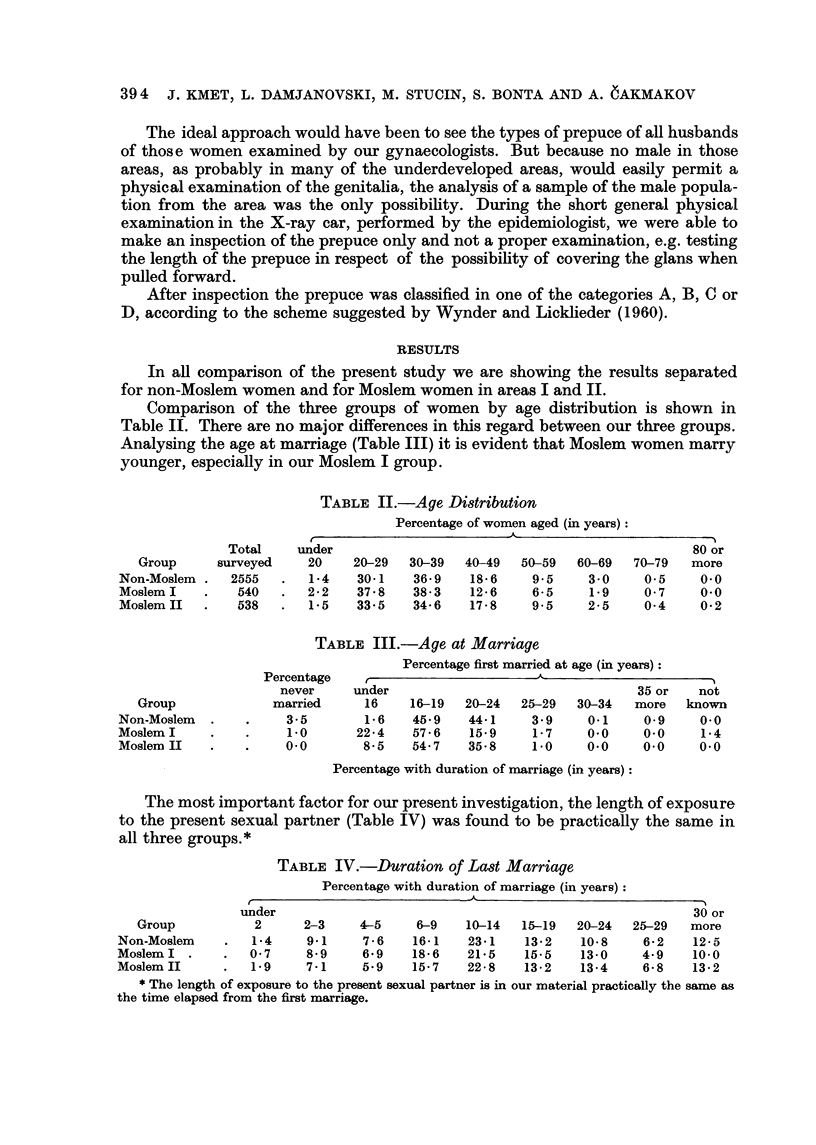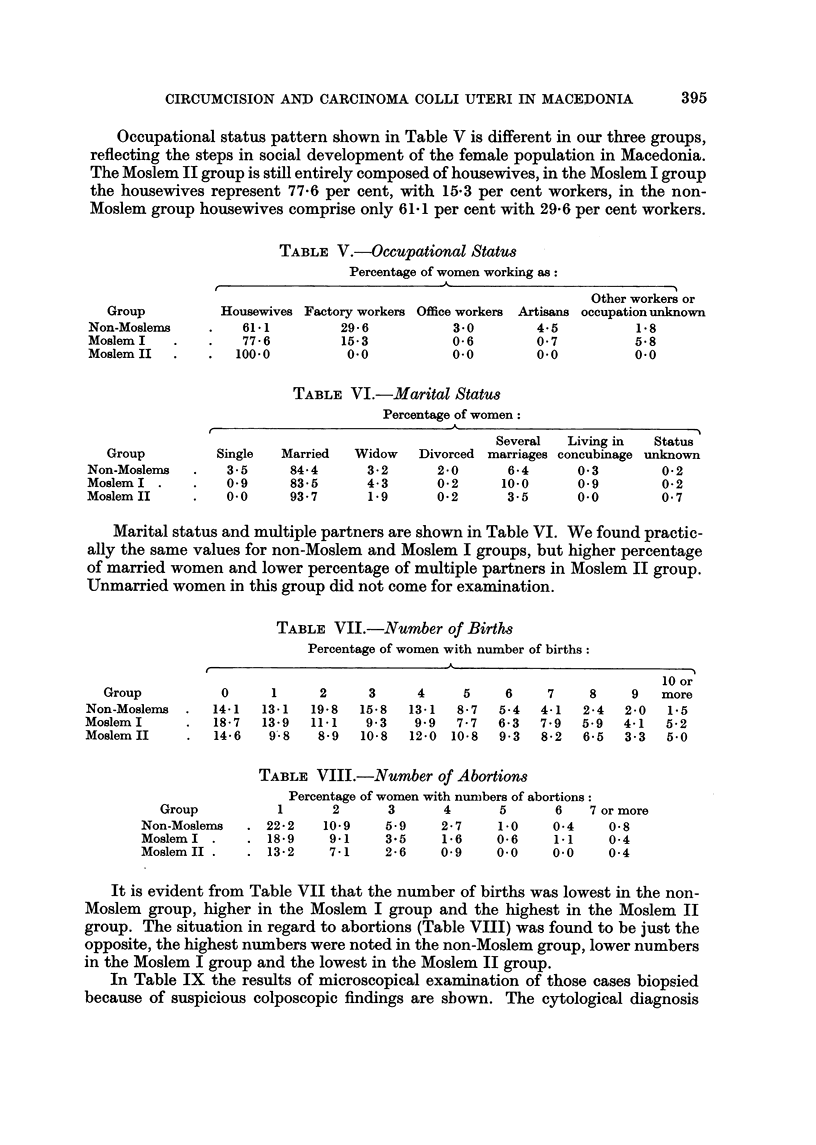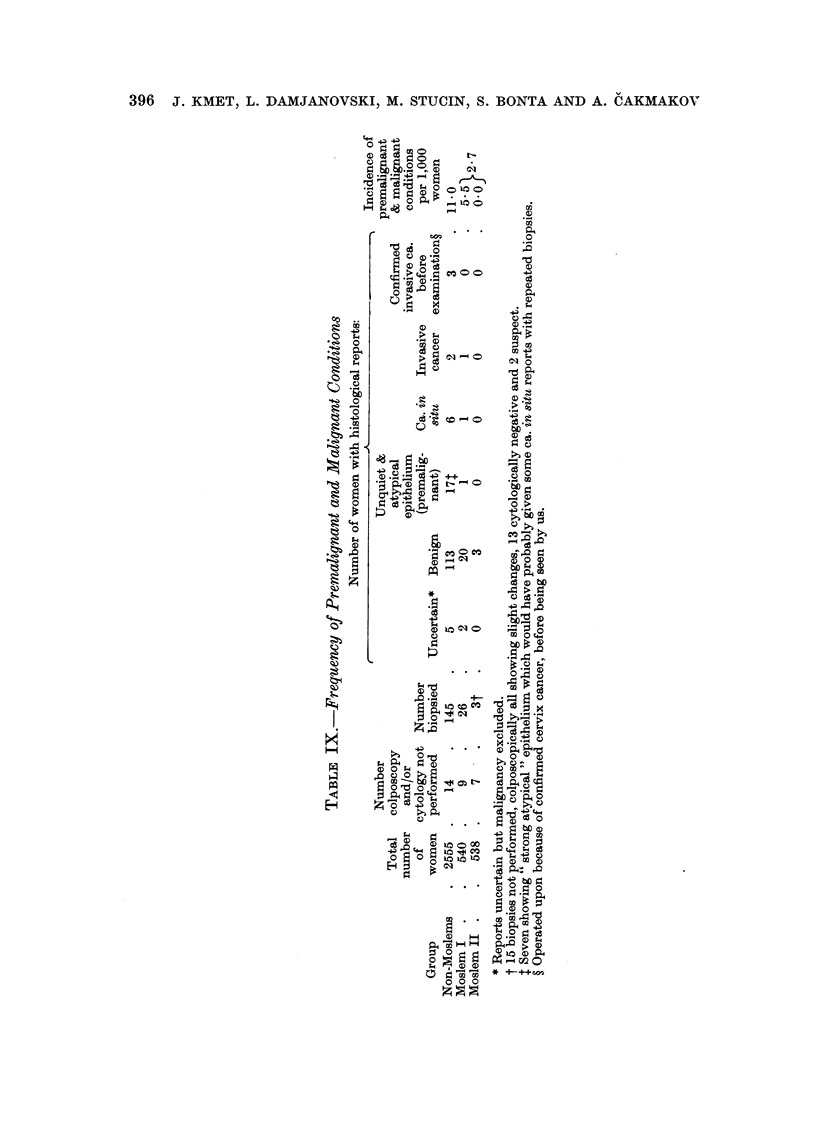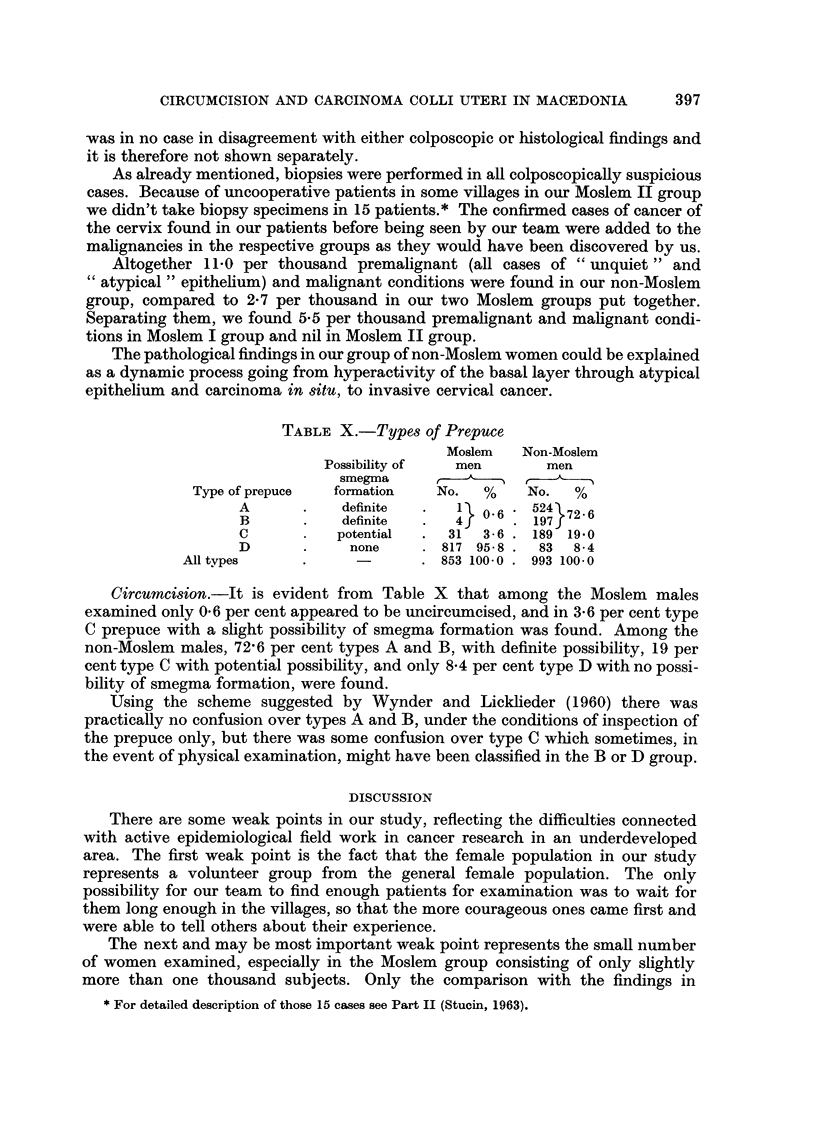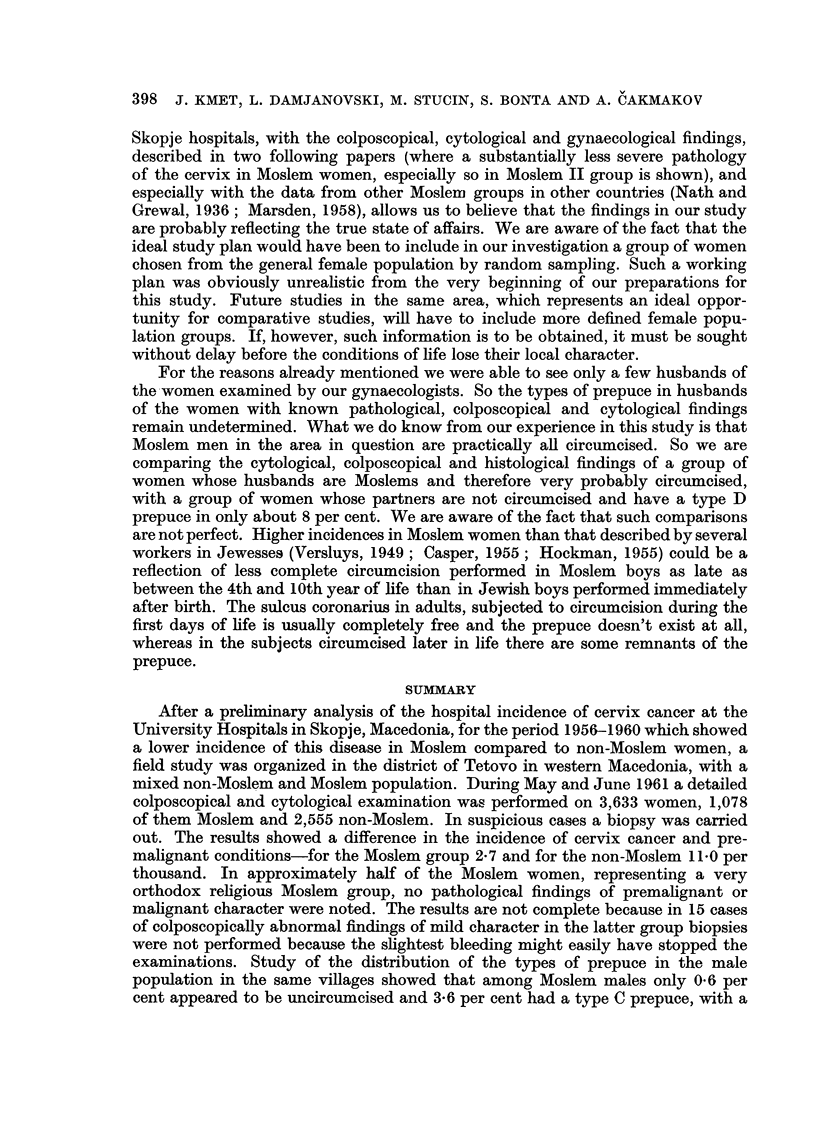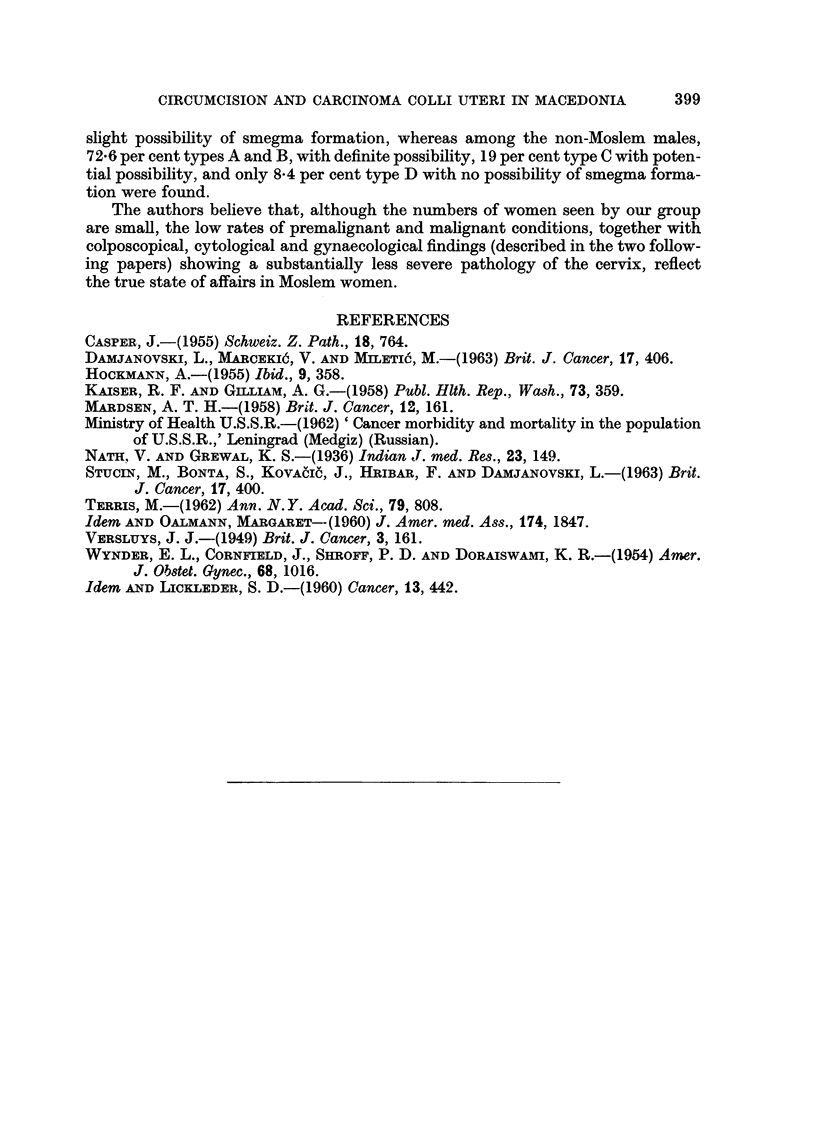# Circumcision and Carcinoma Colli Uteri in Macedonia, Yugoslavia. Results from a Field Study*

**DOI:** 10.1038/bjc.1963.54

**Published:** 1963-09

**Authors:** Janez Kmet, Ljubo Damjanovski, Marija Stucin, Slava Bonta, Anton Cakmakov


### CIRCUMCISION AND CARCINOMA COLLI UTERI IN MACEDONIA, YUGOSLAVIA. RESULTS FROM A FIELD STUDY*

### I. Incidence of Malignant and Premalignant Conditions

JANEZ KMET, LJUBO DAMJANOVSKI, MARIJA STUCIN, SLAVA BONTA AND ANTON ČAKMAKOV

*From the Oncological Institute, Ljubljana ; Hospital Tetovo ; Gynaecological University Hospital, Ljubljana and Gynaecological University Hospital, Skopje, Yugoslavia*

Received for publication May 15, 1963

RECENT meticulous reviews of the epidemiology of cervix cancer (Wynder *et al.*, 1954 ; Kaiser and Gilliam, 1958 ; Terris and Oalmann, 1960 ; Terris, 1962) showed the problem of the relationship of circumcision and cervical cancer to be still open. From all the hypotheses of the possible role of exogenous factors in the aetiology of cervix cancer such as number of pregnancies, infection and cervical lacerations, and of endogenous factors such as hormonal or other biological factors, only the role of coitus seems to be in accordance with all the available epidemiological evidence in this disease. It can explain the very low incidence of cervix cancer in nuns in comparison with the high incidence in prostitutes ; the higher incidence in married women than in single ; the higher incidence in lower income classes where early marriages are common, and the known association of the disease with syphilis.

Several workers have demonstrated the association of cervix cancer and early coitus. They have found that women with onset of the disease at an earlier age have had coitus earlier than those with onset at a later age. These facts, together with the known very low incidence of cervix cancer in Jewesses and the almost complete absence of penile cancer in Jews, have led to the theory of the possible role of smegma as a carcinogenic agent. Many data suggest that cervix cancer is less common among Moslems (Nath and Grewal, 1936; Mardsen, 1958; Ministry of Health U.S.S.R., 1962). Kaiser and Gilliam in their review of the problem write : " reliable data on the incidence of cervix cancer are very badly needed among such groups as the Moslems, Hindus, Bantus, and Fijis. Such rates can now be reliably and relatively inexpensively ascertained through discriminative use of the vaginal cytological technique " (Kaiser and Gilliam, 1958).

Our group decided to investigate this problem in Macedonia, using cytology and colposcopy. The aim of the present study was to show the difference, if any, in the incidence of malignant changes between Moslem and non-Moslem women.

The problem was analysed in a mountainous area of western Macedonia on the Albanian border, the population of which is composed of Macedonians, members of one of the South Slavic national groups who do not practise circumcision, and of Shqyptars† and Turks, national minorities who are Moslems and practise cir-

* Preliminary results were reported in summary form at the 8th International Cancer Congress in Moscow, July 1962.

† A national minority of Albanian origin.

cumcision in male children between approximately the fourth and tenth year of life.

For general orientation in this regard we have made an attempt to show the difference in the hospital incidence of cervix cancer between both religious groups making use of the records of the local hospitals. But the material turned out to be insufficient, for the majority of cases were treated at the University Hospital in Skopje, the capital of Macedonia. Analysing the records in this hospital we have compared the hospital incidence of cervix uteri, corpus uteri, stomach and breast cancer in the female patients of both religious groups, using the number of hospitalizations of Moslem and non-Moslem women as the basis for computation, but each case of malignant tumours being represented in our calculations only once.

We analysed in this way the records of 22,295 hospitalizations, 2,476 in Moslem and 19,819 in non-Moslem women in the Gynecological Hospital and the records of 5,967 hospitalizations, 888 in Moslem and 5,079 in non-Moslem women, in the Surgical Hospital during the years 1956 through 1960.

TABLE I.—*Cancer of Selected Sites Among Female In-patients at the University Hospital, Skopje, Macedonia, 1956–60 ; divided by Religion*

| | | Number of cases among : | | |
|---|---|---|---|---|
| | | Moslems | | |
| Hospital department | Condition | observed | expected* | Non-Moslem observed |
| Gynaecological | Cancer cervix uteri | 18 | 33·7 | 270 |
| | ,, corpus uteri | 4 | 5·2 | 42 |
| | ,, ovary | 3 | 3·5 | 28 |
| | Total in-patients | 2,476 | — | 19,819 |
| Surgical | Cancer breast | 24 | 15·4 | 88 |
| | ,, stomach | 9 | 4·2 | 24 |
| | Total in-patients | 888 | — | 5,079 |

* Calculated on the basis of the proportion of total Moslem to non-Moslem admissions: for example, the expected number of cervix cancer cases in Moslems is $\frac{2,476}{19,819} \times 270 = 33 \cdot 7$.

The results of this analysis are shown in Table I. The observed values for cervix cancer were found to be lower in Moslem, compared to non-Moslem women ; for corpus and ovary cancer the values were the same in both religious groups, as expected. For breast and stomach cancer the values were higher in the Moslem group ; a feature that was not further analysed. The material from the hospitals in Skopje was not uniform. Several cases were not histologically proved and it is possible that some cases classified as cervix cancer, or " carcinoma portionis vaginalis uteri " were of corpus origin or vice versa. Also the age structure of both religious groups was not known to us.

These results, though only approximate, encouraged us to undertake a field study to investigate the circumcision status in the male population in both religious groups and the relationship between this status and the incidence of cervix cancer and premalignant conditions in Moslem and non-Moslem women.

### MATERIALS AND METHODS

A field study was organized during May and June 1961 in villages of the Tetovo district, in a factory in the town of Tetovo and in the town of Gostivar. The female population of these places was invited to be seen by the gynaecologists and the entire population was asked to have a fluorographic X-ray examination, which was a part of the systematic tuberculosis control programme in the area.

An X-ray fluorographic car was included in this investigation to provide an opportunity of inspecting the genitalia in males during a short general physical examination in the car, before the patient was X-rayed. The popular X-ray car helped us also to attract more women for the gynaecological examination.

Our team was composed intentionally of three female gynaecologists, one of them specially trained in colposcopy and one in cytology, the third being used for the general gynaecological examination, an epidemiologist, a nurse, two laboratory technicians and a clerk. Male members of the team were not permitted to enter the rooms where the gynaecological examinations were performed, because Moslem males would resist allowing their wives to be seen by males, although physicians. A form was completed by the nurse for each woman coming for examination, including general information and questions about marital status, number of sexual partners, duration of exposure to the present husband, age at marriage, number of births and abortions and religious practice in the sexual sphere. After the interview the woman was seen by the gynaecologists. The detailed methods of their work are described in Part II of this article (Stucin *et al.*, 1963).

It was extremely difficult to obtain a substantial number of women, especially Moslem women, for this investigation, because of religious prejudices concerning modern medicine and particularly gynaecological examination. Our group had to move from village to village changing the place of work 22 times during the two months of this field study. Rooms for the gynaecological examination were found in local schools and in a few instances only our team shared the benefits of the local outpatient stations.

In the " Moslem " group all women were included who stated that their husbands were Moslems, all others were referred to as non-Moslems. This classification was highly reliable, for mixed marriages between Moslem men and non-Moslem women and *vice versa* are rare exceptions. There were no Jewish subjects in our study.

Our Moslem group is composed of two parts which differ in a very important point in regard to our present problem. The first part (Moslem I) is composed of more emancipated subjects from the towns and some villages who participate in the social events of their villages like their non-Moslem neighbours, a substantial part of them, approximately half, claiming to be of Turkish origin. The second part (Moslem II) belong to the very orthodox Shqyptar Moslem population living in a group of villages in the Tetovo—Gostivar area. They follow very strictly religious practice in sexual life such as washing after intercourse and shaving the genital regions. The social conditions in these villages have changed very little during centuries and the women are often still treated as their husbands' personal property. We found the social life there to be so different from the rest of the Moslem population in the study area, that we decided to treat the women from this group as a separate entity in our analysis.*

*Circumcision.*—We were well informed that Moslems in this region practise circumcision in male children, the so called " sunet " (soonat), but we were not able to obtain any data about the percentage distribution of the circumcised nor about the types of prepuce in noncircumcised men in this area.

* For more details about the characteristics of our ethnic groups see Part III of this paper, written by the Macedonian gynaecologists (Damjanovski *et al.*, 1963).

The ideal approach would have been to see the types of prepuce of all husbands of those women examined by our gynaecologists. But because no male in those areas, as probably in many of the underdeveloped areas, would easily permit a physical examination of the genitalia, the analysis of a sample of the male population from the area was the only possibility. During the short general physical examination in the X-ray car, performed by the epidemiologist, we were able to make an inspection of the prepuce only and not a proper examination, e.g. testing the length of the prepuce in respect of the possibility of covering the glans when pulled forward.

After inspection the prepuce was classified in one of the categories A, B, C or D, according to the scheme suggested by Wynder and Licklieder (1960).

### RESULTS

In all comparison of the present study we are showing the results separated for non-Moslem women and for Moslem women in areas I and II.

Comparison of the three groups of women by age distribution is shown in Table II. There are no major differences in this regard between our three groups. Analysing the age at marriage (Table III) it is evident that Moslem women marry younger, especially in our Moslem I group.

TABLE II.—*Age Distribution*

| Group | Total surveyed | Percentage of women aged (in years): under 20 | 20–29 | 30–39 | 40–49 | 50–59 | 60–69 | 70–79 | 80 or more |
|---|---|---|---|---|---|---|---|---|---|
| Non-Moslem | 2555 | 1·4 | 30·1 | 36·9 | 18·6 | 9·5 | 3·0 | 0·5 | 0·0 |
| Moslem I | 540 | 2·2 | 37·8 | 38·3 | 12·6 | 6·5 | 1·9 | 0·7 | 0·0 |
| Moslem II | 538 | 1·5 | 33·5 | 34·6 | 17·8 | 9·5 | 2·5 | 0·4 | 0·2 |

TABLE III.—*Age at Marriage*

| Group | Percentage never married | Percentage first married at age (in years): under 16 | 16–19 | 20–24 | 25–29 | 30–34 | 35 or more | not known |
|---|---|---|---|---|---|---|---|---|
| Non-Moslem | 3·5 | 1·6 | 45·9 | 44·1 | 3·9 | 0·1 | 0·9 | 0·0 |
| Moslem I | 1·0 | 22·4 | 57·6 | 15·9 | 1·7 | 0·0 | 0·0 | 1·4 |
| Moslem II | 0·0 | 8·5 | 54·7 | 35·8 | 1·0 | 0·0 | 0·0 | 0·0 |

Percentage with duration of marriage (in years) :

The most important factor for our present investigation, the length of exposure to the present sexual partner (Table IV) was found to be practically the same in all three groups.*

TABLE IV.—*Duration of Last Marriage*

| Group | Percentage with duration of marriage (in years): under 2 | 2–3 | 4–5 | 6–9 | 10–14 | 15–19 | 20–24 | 25–29 | 30 or more |
|---|---|---|---|---|---|---|---|---|---|
| Non-Moslem | 1·4 | 9·1 | 7·6 | 16·1 | 23·1 | 13·2 | 10·8 | 6·2 | 12·5 |
| Moslem I | 0·7 | 8·9 | 6·9 | 18·6 | 21·5 | 15·5 | 13·0 | 4·9 | 10·0 |
| Moslem II | 1·9 | 7·1 | 5·9 | 15·7 | 22·8 | 13·2 | 13·4 | 6·8 | 13·2 |

* The length of exposure to the present sexual partner is in our material practically the same as the time elapsed from the first marriage.

Occupational status pattern shown in Table V is different in our three groups, reflecting the steps in social development of the female population in Macedonia. The Moslem II group is still entirely composed of housewives, in the Moslem I group the housewives represent 77·6 per cent, with 15·3 per cent workers, in the non-Moslem group housewives comprise only 61·1 per cent with 29·6 per cent workers.

TABLE V.—*Occupational Status*

| Group | Percentage of women working as: | | | | |
|---|---|---|---|---|---|
| | Housewives | Factory workers | Office workers | Artisans | Other workers or occupation unknown |
| Non-Moslems . | 61·1 | 29·6 | 3·0 | 4·5 | 1·8 |
| Moslem I . . | 77·6 | 15·3 | 0·6 | 0·7 | 5·8 |
| Moslem II . . | 100·0 | 0·0 | 0·0 | 0·0 | 0·0 |

TABLE VI.—*Marital Status*

| Group | Percentage of women: | | | | | | |
|---|---|---|---|---|---|---|---|
| | Single | Married | Widow | Divorced | Several marriages | Living in concubinage | Status unknown |
| Non-Moslems . | 3·5 | 84·4 | 3·2 | 2·0 | 6·4 | 0·3 | 0·2 |
| Moslem I . . | 0·9 | 83·5 | 4·3 | 0·2 | 10·0 | 0·9 | 0·2 |
| Moslem II . | 0·0 | 93·7 | 1·9 | 0·2 | 3·5 | 0·0 | 0·7 |

Marital status and multiple partners are shown in Table VI. We found practically the same values for non-Moslem and Moslem I groups, but higher percentage of married women and lower percentage of multiple partners in Moslem II group. Unmarried women in this group did not come for examination.

TABLE VII.—*Number of Births*

| Group | Percentage of women with number of births: | | | | | | | | | |
|---|---|---|---|---|---|---|---|---|---|---|
| | 0 | 1 | 2 | 3 | 4 | 5 | 6 | 7 | 8 | 9 | 10 or more |
| Non-Moslems . | 14·1 | 13·1 | 19·8 | 15·8 | 13·1 | 8·7 | 5·4 | 4·1 | 2·4 | 2·0 | 1·5 |
| Moslem I . | 18·7 | 13·9 | 11·1 | 9·3 | 9·9 | 7·7 | 6·3 | 7·9 | 5·9 | 4·1 | 5·2 |
| Moslem II . | 14·6 | 9·8 | 8·9 | 10·8 | 12·0 | 10·8 | 9·3 | 8·2 | 6·5 | 3·3 | 5·0 |

TABLE VIII.—*Number of Abortions*

| Group | Percentage of women with numbers of abortions: | | | | | | |
|---|---|---|---|---|---|---|---|
| | 1 | 2 | 3 | 4 | 5 | 6 | 7 or more |
| Non-Moslems . | 22·2 | 10·9 | 5·9 | 2·7 | 1·0 | 0·4 | 0·8 |
| Moslem I . . | 18·9 | 9·1 | 3·5 | 1·6 | 0·6 | 1·1 | 0·4 |
| Moslem II . . | 13·2 | 7·1 | 2·6 | 0·9 | 0·0 | 0·0 | 0·4 |

It is evident from Table VII that the number of births was lowest in the non-Moslem group, higher in the Moslem I group and the highest in the Moslem II group. The situation in regard to abortions (Table VIII) was found to be just the opposite, the highest numbers were noted in the non-Moslem group, lower numbers in the Moslem I group and the lowest in the Moslem II group.

In Table IX the results of microscopical examination of those cases biopsied because of suspicious colposcopic findings are shown. The cytological diagnosis

TABLE IX.—*Frequency of Premalignant and Malignant Conditions*

| Group | Total number of women | Number colposcopy and/or cytology not performed | Number biopsied | Number of women with histological reports: Uncertain* | Benign | Unquiet & atypical epithelium (premalignant) | Ca. *in situ* | Invasive cancer | Confirmed invasive ca. before examination§ | Incidence of premalignant & malignant conditions per 1,000 women |
|---|---|---|---|---|---|---|---|---|---|---|
| Non-Moslems | 2555 | 14 | 145 | 5 | 113 | 17‡ | 6 | 2 | 3 | 11·0 |
| Moslem I | 540 | 9 | 26 | 2 | 20 | 1 | 1 | 1 | 0 | 5·5 } 2·7 |
| Moslem II | 538 | 7 | 3† | 0 | 3 | 0 | 0 | 0 | 0 | 0·0 } 2·7 |

* Reports uncertain but malignancy excluded.

† 15 biopsies not performed, colposcopically all showing slight changes, 13 cytologically negative and 2 suspect.

‡ Seven showing " strong atypical " epithelium which would have probably given some ca. *in situ* reports with repeated biopsies.

§ Operated upon because of confirmed cervix cancer, before being seen by us.

was in no case in disagreement with either colposcopic or histological findings and it is therefore not shown separately.

As already mentioned, biopsies were performed in all colposcopically suspicious cases. Because of uncooperative patients in some villages in our Moslem II group we didn't take biopsy specimens in 15 patients.* The confirmed cases of cancer of the cervix found in our patients before being seen by our team were added to the malignancies in the respective groups as they would have been discovered by us.

Altogether 11·0 per thousand premalignant (all cases of "unquiet" and "atypical" epithelium) and malignant conditions were found in our non-Moslem group, compared to 2·7 per thousand in our two Moslem groups put together. Separating them, we found 5·5 per thousand premalignant and malignant conditions in Moslem I group and nil in Moslem II group.

The pathological findings in our group of non-Moslem women could be explained as a dynamic process going from hyperactivity of the basal layer through atypical epithelium and carcinoma *in situ*, to invasive cervical cancer.

TABLE X.—*Types of Prepuce*

| Type of prepuce | Possibility of smegma formation | Moslem men No. | Moslem men % | Non-Moslem men No. | Non-Moslem men % |
|---|---|---|---|---|---|
| A | definite | 1 | 0·6 | 524 | 72·6 |
| B | definite | 4 | | 197 | |
| C | potential | 31 | 3·6 | 189 | 19·0 |
| D | none | 817 | 95·8 | 83 | 8·4 |
| All types | — | 853 | 100·0 | 993 | 100·0 |

*Circumcision.*—It is evident from Table X that among the Moslem males examined only 0·6 per cent appeared to be uncircumcised, and in 3·6 per cent type C prepuce with a slight possibility of smegma formation was found. Among the non-Moslem males, 72·6 per cent types A and B, with definite possibility, 19 per cent type C with potential possibility, and only 8·4 per cent type D with no possibility of smegma formation, were found.

Using the scheme suggested by Wynder and Licklieder (1960) there was practically no confusion over types A and B, under the conditions of inspection of the prepuce only, but there was some confusion over type C which sometimes, in the event of physical examination, might have been classified in the B or D group.

### DISCUSSION

There are some weak points in our study, reflecting the difficulties connected with active epidemiological field work in cancer research in an underdeveloped area. The first weak point is the fact that the female population in our study represents a volunteer group from the general female population. The only possibility for our team to find enough patients for examination was to wait for them long enough in the villages, so that the more courageous ones came first and were able to tell others about their experience.

The next and may be most important weak point represents the small number of women examined, especially in the Moslem group consisting of only slightly more than one thousand subjects. Only the comparison with the findings in

* For detailed description of those 15 cases see Part II (Stucin, 1963).

Skopje hospitals, with the colposcopical, cytological and gynaecological findings, described in two following papers (where a substantially less severe pathology of the cervix in Moslem women, especially so in Moslem II group is shown), and especially with the data from other Moslem groups in other countries (Nath and Grewal, 1936 ; Marsden, 1958), allows us to believe that the findings in our study are probably reflecting the true state of affairs. We are aware of the fact that the ideal study plan would have been to include in our investigation a group of women chosen from the general female population by random sampling. Such a working plan was obviously unrealistic from the very beginning of our preparations for this study. Future studies in the same area, which represents an ideal opportunity for comparative studies, will have to include more defined female population groups. If, however, such information is to be obtained, it must be sought without delay before the conditions of life lose their local character.

For the reasons already mentioned we were able to see only a few husbands of the women examined by our gynaecologists. So the types of prepuce in husbands of the women with known pathological, colposcopical and cytological findings remain undetermined. What we do know from our experience in this study is that Moslem men in the area in question are practically all circumcised. So we are comparing the cytological, colposcopical and histological findings of a group of women whose husbands are Moslems and therefore very probably circumcised, with a group of women whose partners are not circumcised and have a type D prepuce in only about 8 per cent. We are aware of the fact that such comparisons are not perfect. Higher incidences in Moslem women than that described by several workers in Jewesses (Versluys, 1949 ; Casper, 1955 ; Hockman, 1955) could be a reflection of less complete circumcision performed in Moslem boys as late as between the 4th and 10th year of life than in Jewish boys performed immediately after birth. The sulcus coronarius in adults, subjected to circumcision during the first days of life is usually completely free and the prepuce doesn't exist at all, whereas in the subjects circumcised later in life there are some remnants of the prepuce.

SUMMARY

After a preliminary analysis of the hospital incidence of cervix cancer at the University Hospitals in Skopje, Macedonia, for the period 1956–1960 which showed a lower incidence of this disease in Moslem compared to non-Moslem women, a field study was organized in the district of Tetovo in western Macedonia, with a mixed non-Moslem and Moslem population. During May and June 1961 a detailed colposcopical and cytological examination was performed on 3,633 women, 1,078 of them Moslem and 2,555 non-Moslem. In suspicious cases a biopsy was carried out. The results showed a difference in the incidence of cervix cancer and premalignant conditions—for the Moslem group 2·7 and for the non-Moslem 11·0 per thousand. In approximately half of the Moslem women, representing a very orthodox religious Moslem group, no pathological findings of premalignant or malignant character were noted. The results are not complete because in 15 cases of colposcopically abnormal findings of mild character in the latter group biopsies were not performed because the slightest bleeding might easily have stopped the examinations. Study of the distribution of the types of prepuce in the male population in the same villages showed that among Moslem males only 0·6 per cent appeared to be uncircumcised and 3·6 per cent had a type C prepuce, with a slight possibility of smegma formation, whereas among the non-Moslem males, 72·6 per cent types A and B, with definite possibility, 19 per cent type C with potential possibility, and only 8·4 per cent type D with no possibility of smegma formation were found.

The authors believe that, although the numbers of women seen by our group are small, the low rates of premalignant and malignant conditions, together with colposcopical, cytological and gynaecological findings (described in the two following papers) showing a substantially less severe pathology of the cervix, reflect the true state of affairs in Moslem women.

### REFERENCES

CASPER, J.—(1955) *Schweiz. Z. Path.*, **18**, 764.
DAMJANOVSKI, L., MARCEKIĆ, V. AND MILETIĆ, M.—(1963) *Brit. J. Cancer*, **17**, 406.
HOCKMANN, A.—(1955) *Ibid.*, **9**, 358.
KAISER, R. F. AND GILLIAM, A. G.—(1958) *Publ. Hlth. Rep., Wash.*, **73**, 359.
MARDSEN, A. T. H.—(1958) *Brit. J. Cancer*, **12**, 161.
Ministry of Health U.S.S.R.—(1962) ' Cancer morbidity and mortality in the population of U.S.S.R.,' Leningrad (Medgiz) (Russian).
NATH, V. AND GREWAL, K. S.—(1936) *Indian J. med. Res.*, **23**, 149.
STUCIN, M., BONTA, S., KOVAČIČ, J., HRIBAR, F. AND DAMJANOVSKI, L.—(1963) *Brit. J. Cancer*, **17**, 400.
TERRIS, M.—(1962) *Ann. N.Y. Acad. Sci.*, **79**, 808.
*Idem* AND OALMANN, MARGARET—(1960) *J. Amer. med. Ass.*, **174**, 1847.
VERSLUYS, J. J.—(1949) *Brit. J. Cancer*, **3**, 161.
WYNDER, E. L., CORNFIELD, J., SHROFF, P. D. AND DORAISWAMI, K. R.—(1954) *Amer. J. Obstet. Gynec.*, **68**, 1016.
*Idem* AND LICKLEDER, S. D.—(1960) *Cancer*, **13**, 442.